# Evaluating the effectiveness of genicular radiofrequency ablation for chronic knee pain using the patient-reported outcomes measurement information system (PROMIS) global health-physical health domain: Results of a cross-sectional cohort study

**DOI:** 10.1016/j.inpm.2024.100539

**Published:** 2025-01-09

**Authors:** Todd K. Brown, Marc A. Caragea, Margaret Beckwith, Amelia Ni, Ling Chen, Tyler Woodworth, Michael Blatt, Cole Cheney, Daniel Carson, Keith Kuo, Dustin Randall, Emily Y. Huang, Andrea Carefoot, Megan Mills, Amanda N. Cooper, Allison Glinka Przybysz, Taylor Burnham, Aaron M. Conger, Zachary L. McCormick

**Affiliations:** a School of Medicine, University of Utah, Salt Lake City, UT, USA; b Department of Physical Medicine and Rehabilitation, University of Utah School of Medicine, Salt Lake City, UT, USA; c Division of Biostatistics, Washington University School of Medicine, St Louis, MO, USA; d Mayo Clinic Health System, Mankato, MN, USA; e Johns Hopkins University School of Medicine, Baltimore, MD, USA; f Department of Radiology, University of Utah School of Medicine, Salt Lake City, UT, USA

**Keywords:** Genicular nerve, Knee pain, Radiofrequency ablation, Knee osteoarthritis

## Abstract

**Objective:**

Evaluate the effectiveness of genicular nerve radiofrequency ablation (GNRFA) for chronic knee pain using the Patient-Reported Outcomes Measurement Information System Global Health, Physical Health score (PROMIS-GH-PH).

**Methods:**

Patients who underwent GNRFA at a tertiary academic center were identified by CPT code query and contacted for consent. Demographic, clinical, and procedural characteristics were collected from the electronic medical record of participants with baseline PROMIS-GH-PH scores. Outcome data were collected by standardized telephone survey. Treatment success was defined as a ≥2.3-point improvement in PROMIS-GH-PH score (the minimal clinically important difference [MCID]). Predictor variables of PROMIS score change were evaluated using univariate and multivariate linear regression analyses.

**Results:**

32 patients (65.6 % female; 63.7 ± 10.7 years of age) met eligibility criteria and consented to be included in the study. In this cohort, 56.3 % (18/32; 95 % CI: 37.7–73.6) of patients reported improvement ≥ MCID on PROMIS-GH-PH at a mean follow-up time of 21.5 ± 10.6 months. Linear regression analysis demonstrated that patients who never smoked and those with a Kellgren-Lawrence (KL) osteoarthritis grade of 4 had significantly greater PROMIS-GH-PH score improvements at follow-up compared to current or former smokers and patients with KL grades of 0–3, respectively.

**Conclusion:**

In this real-world cross-sectional cohort study, over 56 % of participants experienced improvment ≥ MCID on PROMIS-GH-PH after undergoing GFRNA for chronic knee pain. Non-smoking status and radiographically severe osteoarthritis were associated with greater PROMIS-GH-PH score improvements from baseline.

## 1 Introduction

Knee osteoarthritis (OA) is a significant source of pain and impaired mobility in older adults, with an estimated 365 million people affected globally [1]. The documented prevalence of chronic knee pain due to knee OA doubled in women and tripled in men between the early 1980’s and the turn of the century, irrespective of changes in BMI and increased lifespan [2], likely due to recognition of the condition and more frequent diagnosis. Total knee arthroplasty (TKA) has demonstrated effectiveness in alleviating pain and restoring function in patients with symptomatic knee OA. Success rates are generally high, varying between 70 and 92 % [[3], [4], [5], [6], [7], [8]], but a subset of patients continue to experience significant pain despite TKA. Furthermore, TKA is not feasible in individuals with various medical comorbidities. Thus, the medical and scientific communities continue to develop and optimize non-surgical alternatives for OA-mediated chronic knee pain, including great interest in genicular nerve radiofrequency ablation (GNRFA) [[9], [10], [11], [12], [13], [14]].

GNRFA is a minimally invasive treatment option used to alleviate chronic knee pain stemming from knee OA as well as persistent pain despite TKA. GNRFA has proven to be a safe and effective pain management modality within these patient populations, importantly, including those who are not suitable candidates for surgery, or prefer to avoid surgical interventions altogether [15].

Historically, linear scales such as the Numeric Rating Scale (NRS) and Visual Analog Scale (VAS) have been employed to measure self-reported changes in pain intensity from baseline in the vast majority of studies that have evaluated GNRFA [13,16]. In addition to pain scores, patients are commonly asked to rate their overall satisfaction with the treatment received using instruments such as the Likert scale or the Patient Global Impression of Change (PGIC). Specific questionnaires have also been developed to assess functional and clinical outcomes in patients with knee OA, such as the Western Ontario McMaster University OA index (WOMAC) and the Knee Injury and Osteoarthritis Outcomes Score for Joint Replacement (KOOS-JR) [17,18]. However, as the use of various questionnaires continues to grow and evolve, a frequently encountered challenge in conducting these intricate assessments of efficacy and efficiency is the issue of respondent fatigue [19].

The National Institutes of Health (NIH) developed the Patient-Reported Outcome Measurement Information System (PROMIS) to alleviate the burden on patients when filling out questionnaires and enhance overall efficiency. This system aims to streamline the process of assessing self-reported health and can be applied across a variety of diseases, unlike disease-specific measures such as the KOOS-JR. The PROMIS Global Health (GH) forms, which include assessments for both Physical Health (PH) and Mental Health, have been evaluated previously in patients experiencing chronic knee pain and individuals undergoing total knee arthroplasty (TKA) [20,21]. Prior research has identified a strong correlation between PROMIS measures and the KOOS-JR assessment tool, which further exemplifies that PROMIS scores effectively capture relevant clinical outcomes in this patient population [22]. A minimal clinically important difference (MCID) of ≥2.3 points has been established for PROMIS-GH-PH, indicating a meaningful threshold for measuring changes in physical health among patients with chronic knee pain [23]. Furthermore, PROMIS metrics more appropriately capture the objective outcomes that prove value, which is defined as the measured health improvement for cost of achieving that improvement [24]. As medical care reimbursement transitions to a value-based system, more widely applicable metrics like the PROMIS-GH-PH will be employed across specialties and treatments, as it can efficiently decrease both administrative burdens on physicians and respondent fatigue for patients.

To our knowledge, no studies have evaluated GNRFA outcomes using PROMIS-GH-PH measures. As such, the objective of this study was to determine the proportion of individuals achieving the MCID (≥2.3-point improvement from baseline) on the PROMIS-GH-PH among patients who received GNRFA as treatment for symptomatic knee OA. Furthermore, we aimed to identify potential demographic and procedural predictors associated with GNRFA success.

## 2 Methods

This study was conducted at a tertiary academic institution with approval from the University of Utah Institutional Review Board (IRB# 138414). The electronic medical record (EMR) was queried using the Current Procedure Technology (CPT) code 64624 to identify patients who underwent GNRFA between May 2017 and February 2021. Patient inclusion criteria were (a) age 18–80 years at the time of GNRFA with (b) a documented diagnosis of knee pain, (c) knee OA confirmed on imaging with Kellgren-Lawrence (KL) classification assigned by a board-certified musculoskeletal radiologist, (d) ≥50 % pain reduction after a single diagnostic genicular nerve block, (e) complete PROMIS-GH-PH scores at baseline prior to GNRFA, and (f) who agreed to participate in a follow-up survey conducted via telephone.

### 2.1 Procedures

All procedures were conducted by physicians specializing in Physical Medicine and Rehabilitation, each with further fellowship training in either Pain Medicine, Sports Medicine, or Interventional Spine and Musculoskeletal Medicine.

#### 2.1.1 Genicular nerve blocks

Patients were supine on a standard fluoroscopy table, with their knees supported and flexed at an angle of approximately 30°. The knee area was prepared in a sterile manner and fluoroscopic guidance was employed for precise needle placement during the diagnostic blocks. Targeted genicular nerves included the superior medial (SMGN), superior lateral (SLGN), and inferior medial genicular nerves (IMGN), with the treating physician optionally selecting additional nerve targets based on the patient’s pain pattern [25]. Anesthesia for the skin and subcutaneous layers above the targeted nerve was administered using 1 % lidocaine. A 25-gauge needle measuring 2.5–3.5 inches in length was then inserted toward each target genicular nerve under fluoroscopic guidance. Target sites were based on the known locations of the genicular nerves as described in anatomic studies [25]. Correct needle positioning was verified through both true lateral and anteroposterior (AP) views and confirmed with the injection of 0.5 mL of iodinated or gadolinium-based contrast medium, except in cases of allergy. Afterwards, 0.5 mL of either 4 % lidocaine or 0.5 % bupivacaine was injected at each targeted site. Upon completion, patients were given a pain log to record their pain levels at 15-min intervals for 6 h post-procedure and instructed to perform activities known to exacerbate their chronic knee pain. A positive block response was defined as ≥50 % reduction of index knee pain.

#### 2.1.2 Radiofrequency ablation

Identical positioning and preparation were used for the GNRFA procedure. Fluoroscopic guidance was utilized to ensure precise needle placement, with the genicular nerve targets being determined based on the prognostic block (and the discretion of the treating physician). The skin and underlying tissues at the target sites were anesthetized using 1 % lidocaine. For cooled GNRFA, a 17-gauge introducer needle was directed to each targeted site [14], followed by the insertion of an 18-gauge probe with a 4-mm active tip (Coolief Cooled Radiofrequency Kit, Halyard Health, Alpharetta, GA). Correct electrode placement was confirmed with lateral and AP fluoroscopic views. Prior to lesioning, 1–2 mL of 2 % lidocaine was injected, and cooled RFA lesions were performed for 165 s at 60 °C. For non-internally-cooled thermal GNRFA, 18-gauge three-tined cannulae with 5-mm active tips (Diros RF Trident, Markham, ON, Canada) or 17-gauge dual-tined cannulae with 10-mm active tips (Nimbus, Stratus Medical, Magnolia, TX) were guided to each target location. Anesthesia with 1–2 mL of 2 % lidocaine was administered before lesioning. Lesions were then performed for 120 s (including a 30-s ramp-up) at 80 °C for each targeted nerve.

### 2.2 Data collection

Patient demographics, clinical characteristics, and procedural variables were collected from the electronic medical record and included age, BMI, duration of pain, gender, smoking status, active opioid prescription for ≥6 months at the time of GNRFA, history of anxiety or depression, baseline PROMIS-GH-PH short form scores, number of nerves targeted during the GNRFA procedure, and radiofrequency ablation probe type. Trained interviewers collected patient-reported outcome data via standardized telephone survey, which consisted of the 10-item PROMIS-GH-PH short form questionnaire shown in Table 1. The primary outcome was the proportion of patients who reported the minimal clinically important difference (MCID) on the PROMIS-GH-PH, defined as an absolute score improvement of ≥2.3 points from baseline [23].

**Table 1:** PROMIS-GH-PH questionnaire.

Item	Question	Scale
1	In general, would you say your health is?	Excellent, very good, good, fair, poor
2	In general, would you say your quality of life is?	Excellent, very good, good, fair, poor
3^a^	In general, how would you rate your physical health?	Excellent, very good, good, fair, poor
4	In general, how would you rate your mental health, including your mood and your ability to think?	Excellent, very good, good, fair, poor
5	In general, how would you rate your satisfaction with your social activities and relationships?	Excellent, very good, good, fair, poor
6^a^	To what extent are you able to carry out your everyday physical activities such as walking, climbing stairs, carrying groceries, or moving a chair?	Completely, mostly, moderately, a little, not at all
7^a^	How would you rate your pain on average?	0-10 NRS with 0 representing no pain and 10 being the worst pain imaginable
8^a^	How would you rate your fatigue on average?	None, mild, moderate, severe, very severe
9	In general, please rate how well you carry out your usual social activities and roles.	Excellent, very good, good, fair, poor
10	How often have you been bothered by emotional problems such as feeling anxious, depressed, or irritable?	Never, rarely, sometimes, often, always

NRS = numeric rating scale; PROMIS-GH-PH = Patient Reported Outcomes Measurement Information System Global Health-Physical Health.

a Physical Health subscale score is calculated by summing responses to questions 3, 6, 7, and 8.

### 2.3 Statistical analysis

Participant demographics and clinical characteristics were summarized using descriptive statistics, with calculated means/standard deviations (SD) for continuous variables, and frequencies/percentages for categorical variables. PROMIS-GH-PH change scores (post-GNRFA score minus pre-GNRFA score) were calculated and normality was confirmed (Shapiro-Wilk test *p* = 0.81). Univariate linear regression analysis examined the association between PROMIS-GH-PH change scores and select predictor variables, including KL grade, number of GNRFA lesions, diagnosed depression or anxiety, opioid use at time of GNRFA, smoking status, BMI, and duration of pain. Predictor variables with *p* < 0.20 according to univariate analysis were included in the multivariate regression analysis as covariates. A backwards selection method was applied to the initial “full” multivariate regression model via stepwise removal of variables with the largest non-significant *p*-values until only significant predictor variables remained. Least squares means and 95 % confidence intervals (CI) between groups were estimated from the final multivariate linear regression model. Differences between pre- and post-GNRFA PROMIS-GH-PH scores were analyzed using a paired *t*-test. All statistical tests were two-sided and *p*-values <0.05 were considered statistically significant.

## 3 Results

A total of 32 participants met eligibility criteria and were included in the analysis. Participant demographics and clinical characteristics are presented in Table 2, Table 3. In this cohort, 65.6 % (21/32) of participants were female. Participants had an average knee pain duration of 4.7 ± 3.2 years prior to undergoing GNRFA. Over half of the study cohort (53.1 %; 17/32) had a worst compartment KL grade of 4; the remaining participants had KL grades of 0–3. The average participant age was 63.7 ± 10.7 years (range 35–77 years) with an average BMI of 32.2 ± 7.38 kg/m^2^ 62.5 % of participants had a history of anxiety and/or depression. 28.0 % of participants admitted to being former smokers, and 3.1 % of participants identified as current smokers at the time of their GNRFA procedure. 34.4 % of the study participants were consuming opioids at the time of GNRFA, and this decreased to 15.6 % post-GNRFA when PROMIS-GH-PH scores were collected at follow-up. With regard to the number of lesions, 15.6 % (5/32) underwent the “classic” 3-lesion GNRFA protocol, while the majority of participants (84.3 %, 27/32) received more than 3 lesions during the procedure. Outcome data were collected from participants at a mean follow-up time of 21.5 ± 10.6 months (range 10–40 months) post-GNRFA. Mean PROMIS-GH-PH score improvement from baseline was 2.59 points, with 56.3 % (18/32; 95 % CI: 37.7–73.6) of participants reporting improvements that met or exceeded the MCID of ≥2.3 points (Table 4).

**Table 2:** Participant demographics, clinical characteristics, and procedure-related variables (*N* = 32; categorical variables).

Categorial Variable	No.	%
**Gender**		
Male	11	34.4
Female	21	65.6
**Smoking**		
Never	22	68.9
Current	1	3.1
Former	9	28.0
**Opioid use (baseline)**		
Yes	11	34.4
No	21	65.6
**Opioid use (follow-up)**		
Yes	5	13.2
No	27	86.8
**History of anxiety and/or depression**		
Yes	20	62.5
No	12	37.5
**KL grade**		
0-3	15	46.9
4	17	53.1
**Number of GNRFA lesions**		
Conventional 3	5	15.6
> 3	27	84.4

GNRFA = genicular nerve radiofrequency ablation; KL = Kellgren-Lawrence; PROMIS-GH-PH = Patient Reported Outcomes Measurement Information System Global Health-Physical Health.

**Table 3:** Participant demographics, clinical characteristics, and procedure-related variables (*N* = 32; continuous variables).

Continuous Variable	Mean	SD	Median	Min	Max
Age (years)	63.7	10.7	63.5	35.0	77.0
BMI (kg/m^2^)	32.2	7.4	36.0	24.0	54.0
Duration of pain (years)	4.7	3.2	4.0	0.33	15.0
PROMIS-GH-PH score					
Pre-GNRFA (baseline)	37.4	7.5	36.0	24.0	54.0
Post-GNRFA (follow-up)	40.0	9.2	40.0	20.0	62.0
Change (follow-up minus baseline)	2.59	8.0	4.0	−17.0	21.0

Max = maximum value; Min = minimum value; PROMIS-GH-PH = Patient Reported Outcomes Measurement Information System Global Health-Physical Health; SD = standard deviation.

**Table 4:** Proportion of participants reporting the MCID on PROMIS-GH-PH at follow-up (*N* = 32).

Outcome	No. (%)	95 % CI
≥2.3-point PROMIS-GH-PH improvement		
Yes	18 (56.3)	37.7, 73.6
No	14 (43.8)	26.4, 62.3

CI = confidence interval; MCID = minimal clinically important difference; PROMIS-GH-PH = Patient Reported Outcomes Measurement Information System Global Health-Physical Health.

According to univariate linear regression analysis, only KL grade (*p* = 0.02) and smoking status (*p* = 0.02) demonstrated statistically significant correlations with PROMIS-GH-PH score change (Table 5). Individuals with a worst compartment KL grade of 4 (*N* = 17) had a mean PROMIS-GH-PH score improvement of 5.71 ± 6.32 points, while those with KL grades of 0–3 (*N* = 15) reported worse PROMIS-GH-PH scores by a mean of −0.93 ± 8.50 points. Similarly, those who had never smoked (*N* = 22) reported an average PROMIS-GH-PH score improvement by a mean of 4.77 ± 6.40 points, while those who were either former or current smokers (*N* = 10) experienced worsened PROMIS-GH-PH scores by an average of −2.20 ± 9.46 points.

**Table 5:** Results of univariate and multivariate linear regression analyses examining associations between PROMIS-GH-PH change score and select predictor variables.

Predictor	Univariate		Multivariate			
			Full model		Final model	
	*Β*^a^	*p*	*Β*^a^	*p*	*Β*^a^	*p*
**Worst KL grade** (0–3 vs. 4)	+6.64	**0.02**^b^	+5.57	**0.04**	+5.67	**0.03**
**Number of lesions** (3 vs. >3)	+6.63	0.09^b^	+1.14	0.78	–	–
**Depression** (yes vs. no)	−1.98	0.50	–	–	–	–
**Anxiety** (yes vs. no)	−0.34	0.91	–	–	–	–
**Opioid use** (yes vs. no)	+1.04	0.74	–	–	–	–
**Smoking** (never vs. current or former)	−6.97	**0.02**^b^	−4.98	0.12	−5.89	**0.04**
**BMI** (for an increase of 1 kg/m^2^)	+0.26	0.20^b^	+0.19	0.28	–	–
**Pain duration** (for an increase of 1 year)	+0.25	0.59	–	–	–	–

KL = Kellgren-Lawrence; PROMIS-GH-PH = Patient Reported Outcomes Measurement Information System Global Health-Physical Health.

Bold values denote statistical significance at *p* < 0.05.

a From linear regression model: represents the difference in mean PROMIS-GH-PH change scores between the factors of a predictor variable.

b Predictors with *p* < 0.20 according to univariate linear regression analysis were included as covariates in the full multivariate regression model.

An initial multivariate linear regression model was constructed using the following predictors with *p* < 0.20 from the univariate analysis: KL grade, number of GNRFA lesions, smoking status, and BMI (Table 5). After backwards selection to systematically remove variables with the largest non-significant *p*-values, only KL grade (*p* = 0.03) and smoking status (*p* = 0.04) remained in the final model as statistically significant predictor variables (Table 5). Least square means and 95 % confidence intervals from the final multivariate model are presented in Table 6. According to these estimates, the mean PROMIS-GH-PH score improvement among participants with a worst compartment KL grade of 4 was 4.15 (95 % CI: 0.38–7.91) points and 4.26 (95 % CI: 1.17–7.34) points for nonsmokers. We also tested for an interaction between KL grade and smoking but did not find it to be significant (*p* = 0.22).

**Table 6:** Least square means estimated from final multivariate linear regression model.

Predictor	LS Mean	95 % CI
Worst KL Grade		
0–3	−1.52	−5.26, 2.21
4	4.15	0.38, 7.91
Smoking		
Never smoked	4.26	1.17, 7.34
Current or former smoker	−1.63	−6.18, 2.92

CI = confidence interval; LS = least square.

A paired *t*-test revealed no statistically significant difference between the pre- and post-GNRFA PROMIS-GH-PH scores (t-score 1.83; *p* = 0.07). In addition, we performed an exploratory sub-analysis of the proportion of individuals who reported a PROMIS-GH-PH score improvement by > MCID, stratified by various categories of pain relief following prognostic genicular nerve blocks (50–79 %, 80–99 %, and 100 % relief) of concordant duration to the local anesthetic used. This analysis demonstrated no statistically significant differences between categories of relief thresholds associated with the prognostic genicular nerve block (t-score 1.70; p = 0.23). No intergroup differences were demonstrated.

## 4 Discussion

In this study, we assessed the effectiveness of GNRFA as a treatment for symptomatic knee OA in a real-world population using the Patient-Reported Outcomes Measurement Information System Global Health, Physical Health score (PROMIS-GH-PH) Domain. To our knowledge, this is the first reported use of PROMIS-GH-PH to evaluate and validate GNRFA treatment outcomes. Our findings indicate that mean PROMIS-GH-PH score improvement from baseline was 2.59 points in this cohort, with 56.3 % of patients reporting improvements that met the MCID of ≥2.3 points at an average follow-up of 21.5 ± 10.6 months post-GNRFA. Additionally, similar to other GNRFA literature, we found that KL grade and smoking status are predictors for improving treatment outcomes using PROMIS-GH-PH scores [26]. For individuals with a KL grade of 4, the mean improvement in PROMIS-GH-PH score was 4.15 points. For those who never smoked, mean PROMIS-GH-PF score improvement was 4.26 points. Anxiety or depression, opioid use, duration of pain, BMI, and the number of RF lesions were not statistically significant predictors of PROMIS-GH-PH score improvement after GNRFA.

In contrast to our findings, the landmark 2021 paper by Chen et al. did not identify KL grade as a significant predictor of treatment success [27]. However, it is important to note that the investigators stratified patients into groups of KL grades 1–2 vs. 3–4, whereas patients in the present study were grouped according to KL grades 0–3 vs. 4. In our study, a KL grade of 4 was significantly associated with a positive treatment response, which aligns with recent GNRFA literature [26]. Chen et al. also found no association between smoking status and treatment success, while we observed that GNRFA treatment outcomes were significantly improved among nonsmokers [27]. Another factor that could explain the differences between our results and those of Chen et al. are the outcome measures used. While Chen et al. defined success as ≥30 % pain reduction for at least 3 months, we employed PROMIS-GH-PH scores, thereby assessing different aspects of patient wellbeing and responsiveness to GNRFA. Conversely, in a separate study, House et al. found that both shorter duration of knee pain and a KL grade below 4 were linked to successful treatments when using cooled radiofrequency [28]. These discrepancies might be due to our smaller sample size, or the different outcome measurements employed.

The findings of this study offer compelling evidence that GNRFA serves as an effective treatment for individuals with symptomatic knee OA as measured by PROMIS-GH-PH. The notable long-term improvement in PROMIS-GH-PH scores indicate that GNRFA has the potential to provide durable, clinically significant improvements to the physical health of patients. Furthermore, the results highlight the importance of considering patient-specific factors that may impact success, such as KL grade and smoking status. Such considerations may aid clinicians in making more personalized, and thereby more effective, treatment recommendations for patients with painful knee OA.

### 4.1 Limitations

When interpreting the results of our study, it is important to be aware of its limitations. The data comes from a single institution, potentially affecting the generalizability of the findings to other populations due to institution-specific variables. The small study sample size reduces confidence in the certainty of the findings. In particular, our exploratory sub-analysis of responders based on the MCID definition for PROMIS-GH-PH score improvement stratified by percentage relief following prognostic genicular nerve blocks was underpowered. A larger study is needed to determine whether various categories of percentage pain relief associated with a genicular nerve block are predictive of clinical outcomes as assessed by the PROMIS-GH-PH domain. Notably, we previously found that a 50 % pain relief threshold following genicular nerve blocks does not predict a clinically meaningful response in pain reduction following genicular nerve RFA when compared to not using a genicular nerve block to select patients for genicular nerve RFA [29]. However, similar exploratory sub-analysis did show a trend in which higher thresholds of pain relief associated with the genicular nerve block (i.e. 80 %, 100 %) were associated with higher responder rates based on 50 % reduction in index knee pain following genicular nerve RFA. It is notable that this trend was present despite a less comprehensive genicular nerve RFA lesioning protocol in that study. The present study also contains limitations inherent to most observational studies featuring multiple proceduralists, including gaps in data, inconsistent reporting, and possible variations in procedural technique. Alternatively, this does improve generalizability. Various hidden confounding factors could affect our conclusions. Furthermore, given the pragmatic nature of this study, we did not monitor or control for co-interventions, which could have a bearing on the actual effectiveness of GNRFA.

## 5 Conclusion

Given the escalating economic and societal burden of chronic knee pain, non-surgical treatments such as GNRFA are becoming increasingly important for improving pain and function in patients with symptomatic knee OA. This study is the first to report PROMIS-GH-PH scores assessing GNRFA outcomes, revealing that 56.3 % of patients experienced improvements that met or exceeded the established MCID of 2.3 points at a mean follow-up time of almost 2 years after GNRFA.

## Funding

The Skaggs Foundation for Research.

## Declaration of competing interest

The authors declare the following financial interests/personal relationships which may be considered as potential competing interests: Zachary McCormick reports financial support was provided by The Skaggs Foundation for Research. Zachary McCormick reports a relationship with International Pain & Spine Intervention Society that includes: board membership. Zachary McCormick reports a relationship with Avanos Medical Inc that includes: consulting or advisory and funding grants. Zachary McCormick reports a relationship with 10.13039/100008497Boston Scientific Corporation that includes: funding grants. Zachary McCormick reports a relationship with 10.13039/100019095Relievant Medsystems Inc that includes: funding grants. Zachary McCormick reports a relationship with Saol Therapeutics that includes: consulting or advisory and funding grants. Zachary McCormick reports a relationship with Spine Biopharma that includes: funding grants. Zachary McCormick reports a relationship with 10.13039/100019735SPR Therapeutics Inc that includes: funding grants. Zachary McCormick reports a relationship with Stratus Medical that includes: funding grants. Zachary McCormick reports a relationship with Stryker that includes: consulting or advisory. Zachary McCormick reports a relationship with OrthoSon that includes: consulting or advisory. Taylor Burnham reports a relationship with Diros Technology Inc that includes: funding grants. Taylor Burnham reports a relationship with Avanos Medical Inc that includes: consulting or advisory. Aaron Conger reports a relationship with Stratus that includes: funding grants. If there are other authors, they declare that they have no known competing financial interests or personal relationships that could have appeared to influence the work reported in this paper.
